# The role of vaccine status homophily in the COVID-19 pandemic: a cross-sectional survey with modelling

**DOI:** 10.1186/s12889-024-17957-5

**Published:** 2024-02-14

**Authors:** Elisha B. Are, Kiffer G. Card, Caroline Colijn

**Affiliations:** 1 Mathematics, Simon Fraser University, Burnaby, BC Canada; 2 Pacific Institute On Pathogens, Pandemics and Society (PIPPS), Simon Fraser University, Burnaby, BC Canada; 3 Faculty of Health Sciences, Simon Fraser University, Burnaby, BC Canada; 4 Institute for Social Connection, Victoria, BC Canada

**Keywords:** COVID-19, Vaccine, Homophily, Contact network, Mathematical model, Transmission

## Abstract

**Background:**

Vaccine homophily describes non-heterogeneous vaccine uptake within contact networks. This study was performed to determine observable patterns of vaccine homophily, as well as the impact of vaccine homophily on disease transmission within and between vaccination groups under conditions of high and low vaccine efficacy.

**Methods:**

Residents of British Columbia, Canada, aged ≥ 16 years, were recruited via online advertisements between February and March 2022, and provided information about vaccination status, perceived vaccination status of household and non-household contacts, compliance with COVID-19 prevention guidelines, and history of COVID-19. A deterministic mathematical model was used to assess transmission dynamics between vaccine status groups under conditions of high and low vaccine efficacy.

**Results:**

Vaccine homophily was observed among those with 0, 2, or 3 doses of the vaccine. Greater homophily was observed among those who had more doses of the vaccine (*p* < 0.0001). Those with fewer vaccine doses had larger contact networks (*p* < 0.0001), were more likely to report prior COVID-19 (*p* < 0.0001), and reported lower compliance with COVID-19 prevention guidelines (*p* < 0.0001). Mathematical modelling showed that vaccine homophily plays a considerable role in epidemic growth under conditions of high and low vaccine efficacy. Furthermore, vaccine homophily contributes to a high force of infection among unvaccinated individuals under conditions of high vaccine efficacy, as well as to an elevated force of infection from unvaccinated to suboptimally vaccinated individuals under conditions of low vaccine efficacy.

**Interpretation:**

The uneven uptake of COVID-19 vaccines and the nature of the contact network in the population play important roles in shaping COVID-19 transmission dynamics.

**Supplementary Information:**

The online version contains supplementary material available at 10.1186/s12889-024-17957-5.

## Introduction

COVID-19 is a respiratory illness caused by severe acute respiratory syndrome coronavirus 2 (SARS-CoV-2), which is transmitted predominantly via aerosols and droplets [1]. In high-income countries, the general population case fatality rate of COVID-19 is sufficiently high to necessitate widespread public health interventions and targeted protections for vulnerable populations, such as seniors and people who are immunocompromised [2].

Fortunately, several safe and effective vaccines are available that can prevent severe COVID-19 and reduce mortality risk, although they have lower effectiveness against transmission than initially hoped [3]. At the individual level, the effectiveness of these vaccines wanes over time, and is subject to immune escape [4]. At the population level, the effectiveness of these vaccines is also dependent on their uptake within and across geographic regions and social networks [5, 6]. Of course, vaccine uptake is heterogeneous within any given population, and this heterogeneity may create disproportionate risk for SARS-CoV-2 transmission within and across communities.

Vaccine hesitancy is an important factor shaping vaccine uptake [7]. A 2014 systematic review documented a range of factors that influence vaccine hesitancy, including contextual influences (e.g., politics, government, religion, geographic patterns, media); individual and social group influences (e.g., beliefs, attitudes, knowledge, trust in healthcare systems and providers); and vaccine-specific issues (e.g., mode of administration and delivery, vaccination schedules, risk vs. benefit) [8]. The results showed that vaccine status tends to cluster with sociodemographic characteristics, such as age, socioeconomic status, race/ethnicity, and political orientation [8–10].

Homophily is a principle in sociology and mathematical modelling that describes the clustering of individual-level characteristics, such as vaccination status, within social networks [11]. Kadelka and McCombs [12] suggested that vaccine homophily may impact COVID-19 vaccine effectiveness given the potential for uneven vaccination uptake. Modelling studies have explored the impact of homophily in a range of contexts, and its impact on transmission dynamics is well documented [13, 14]. For instance, one modelling study argued that the mixing of vaccinated and unvaccinated groups contributes to a considerable risk of infection for the vaccinated group, occurring at a rate that is disproportionately higher than what would be expected based solely on the contact between the two groups [15]. However, these previous studies were not based on descriptive data regarding vaccine homophily. Broadly, empirical research related to vaccine homophily in the context of the COVID-19 pandemic has been limited. Therefore, it is important to describe COVID-19 vaccine homophily and its relationship to vaccination status to gain an improved understanding of COVID-19 transmission [16–19].

The present study was performed to characterize observable patterns of vaccine homophily and to determine the impact of vaccine homophily on COVID-19 transmission within and between vaccination groups under conditions of high and low vaccine efficacy. In doing so, this paper makes important contributions by connecting vaccination and contact heterogeneity, which are two crucial determinants of transmission dynamics and assesses the impacts of these determinants under low and high vaccination efficacy scenarios.

## Methods

### Participant recruitment

**Fig. 1:**
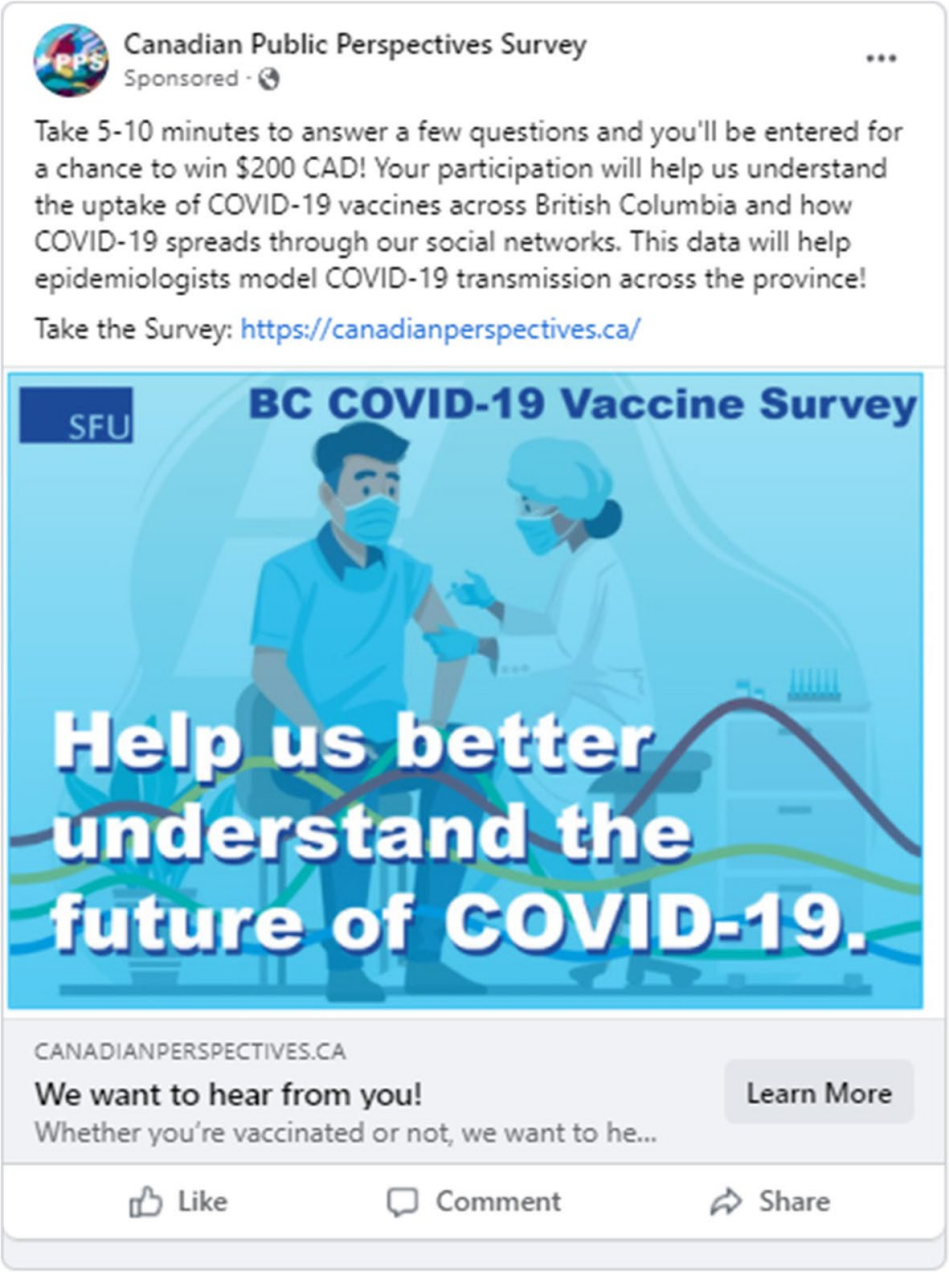
Example meta advertisement used for participant recruitment

Participants were recruited using paid Facebook and Instagram advertisements (Fig. 1) between February 16, 2022, and March 3, 2022, a period during which the average number of new COVID-19 cases in British Columbia, Canada was declining (7-Day Rolling Average: 865 on February 16, 487 on March 3) and the province continued to experience high numbers of Omicron variant infections [20].

### Data collection

After providing informed consent, potential participants recruited via Facebook and Instagram advertisements were screened for eligibility. The eligibility criteria restricted participation to individuals aged 16 years or older living in British Columbia (BC), Canada. Participants completed an online survey delivered in English using the Qualtrics platform, which assessed participants’ history of COVID-19, the extent to which they were following provincial mandates and guidelines for COVID-19 prevention, and how many COVID-19 vaccine doses they had received. Participants also reported on the perceived COVID-19 history of their regular contacts, the perceived level of compliance to COVID-19 prevention guidelines and mandates among regular contacts, the vaccination status of their household and non-household contacts, and the number of household and non-household contacts with whom they had recent contacts. Supplemental Table S1 provides an overview of how these variables were measured by providing the question text and response options.

Additionally, the following demographic data of the participants were collected: age (numerical), gender (Male; Female; Non-binary), ethnicity (African, Caribbean, or Black; Arab or West Asia; East Asian; Indigenous; Latin American; South Asian; Southeast Asian; White; Other), education level (Some high school; High school diploma or equivalent; Some college or trades training; Some university; College or trades certificate or diploma; University degree or higher), annual household income ($0 to $150,000 or higher), and whether participants were born in Canada (Yes; No, moved to Canada in the last 5 years; No, moved to Canada more than 5 years ago). Postal code was also assessed and was used to assign participants to one of the 5 regional health authorities in BC.

### Data analysis

#### Aim 1. Characterizing vaccine homophily and analyzing

##### Its association with COVID-19 risk factors and prevention behaviours

To characterize observable patterns of vaccine homophily, self-reported vaccination status, COVID-19 prevention behaviours, contact network size, and self-reported COVID-19 infection, descriptive analyses of survey responses were conducted in R version 4.1.3. [21]. Data were cleaned using the *Tidyverse* collection of R packages [22]. As a preliminary step, participants with missing data on demographic-variables (i.e., age, gender, ethnicity, income, education level, immigration status, and health authority) or poor-quality responses (i.e., those in which incongruent responses were provided across questions, indicating imprecise answering) were removed from the analysis. Removal of data with missing demographic variables was done because our sample weighting procedure was not tolerant of missing data [23]. The remaining observations were weighted by weighting variables using iterative proportional fitting raking estimation, which is a well-established approach for multivariable weighting when only the marginal proportions for each variable are known [24–26]. Raking estimation was implemented using the *anesrake* package [27] with marginal proportions for each weighting variable derived from the 2016 Canadian Census Profile for BC [28]. The *survey* package was used to generate weighted descriptive statistics [29]. Table 1 provides target weights used in weighting.

**Table 1:** Target weights

**Demographic**	**Target %**
**Age**	
29 or younger	0.215
30–39	0.153
40–49	0.156
50–59	0.179
60–69	0.155
70 or older	0.142
**Gender**	
Men	0.485
Women	0.505
Non-binary	0.010
**Ethnicity**	
White	0.637
East Asian	0.166
South and Southeast Asian	0.092
Indigenous	0.059
Other	0.046
**Educational Attainment**	
Some Post-Secondary Training	0.304
High School Diploma or Less	0.450
University Degree	0.246
**Household Income**	
Less than $30,000	0.189
$30,000 to $59,999	0.240
$60,000 to $89,999	0.195
$90,000	0.376
**Born in Canada**	
No	0.305
Yes	0.695
**Health Authority**	
Fraser	0.368
Interior	0.157
Island	0.164
Northern	0.062
Vancouver Coastal	0.249

Weighted descriptive data were plotted using the *questionr* and *ggplot2* packages [30, 31]. For descriptive statistics, all observations were included in the weighted sample without removing them due to non-response (e.g., early survey drop off, refusal to answer). This allowed us to make best use of the data available. All variables had less than 5% missingness.

To understand clustering between risk factors for COVID-19 and participant’s self-reported vaccination status, the chi-square and Kruskal–Wallis tests were used to compare participants with 0, 1, 2, or 3 or more doses with regard to select COVID-19 related personal and network characteristics. Post-hoc pairwise comparisons were also conducted to examine the differences in network size across different vaccine dose categories (i.e., Zero doses, One dose, Two doses, Three doses) using the Wilcoxon rank sum test with a continuity correction. In these additional analyses, the Benjamini-Hochberg procedure was employed for adjusting *p*-values to control for the false discovery rate in multiple comparisons.

The following variables are determined based on the outcomes of the survey:

- ***Average Number of Doses Among Contacts.*** The average numbers of doses among household, non-household, and overall contacts were calculated using self-reported estimates of the number of doses that participants believed each of their contacts had received.
- ***Proportion of Contacts with* ≥ *1 Dose.*** The proportion of contacts with ≥ 1 dose was calculated using self-reported data on the number of doses that participants believed each of their contacts had received.
- ***Prevalence adjusted Vaccine Homophily.*** As homophily estimates depend on the prevalence of each vaccination group, we calculated prevalence adjusted homophily (PAH) using a method proposed in [32]. We define prevalence-adjusted vaccine homophily ${h}_{i}$ as follows:

1 $${h}_{i}=\left\{\begin{array}{cc}\frac{{\delta }_{i}-{q}_{i}}{1-{q}_{i}}& {\text{if}}\, {\delta }_{i}\ge {q}_{i},\\ \frac{{\delta }_{i}-{q}_{i}}{{q}_{i}}& {\text{if}}\, {\delta }_{i}<{q}_{i},\end{array}\right.$$

where ${\delta }_{i}=\frac{{w}_{i} + {d}_{i}}{{\sum }_{j=0}^{3}({w}_{j} + {d}_{j})},$ which represents the proportion of contacts in an individual’s network that are of the same vaccination status. Here, ${q}_{i}$ represents the prevalence of vaccination status $i$ which is calculated from vaccination uptake data in BC during the study period [33]. The term ${w}_{i}$ denotes the number of people in the contact network of an individual with $i$ doses who have also received $i$ doses, while ${d}_{i}$ represents the number of people in the household of an individual with $i$ doses who have received $i$ doses themselves. Each participant, therefore, has their own ${h}_{i}$ value. To validate the mathematical expression for ${h}_{i}$, we consider three special cases:

Firstly, ${\delta }_{k}=1$ for the ${k}^{th}$ individual, possible only if that individual only contacts those in their group. From Eq. (1), it is easy to show that in this case ${h}_{k}= 1$. This implies perfect homophily.

Secondly, if ${\delta }_{k}$ = 0 for the ${k}^{th}$ individual, then none of the contacts are with people of the same vaccination status. In this case, ${h}_{k}= -1$, which implies perfect heterophily.

Thirdly, if the ${k}^{th}$ individual does not have mixing preferences and prefers to mix randomly between vaccination groups, we can expect ${\delta }_{k}$ to be approximately the prevalence of the ${k}^{th}$ group, implying ${\delta }_{k}={q}_{k}$, which yields ${h}_{k}=0$. We have ${h}_{i} \in [-\mathrm{1,1}]$ for all $i$, with -1 and 1 at the appropriate extremes and 0 reflecting no contact preference (by vaccination status). In summary, the PAH score is calculated by subtracting the prevalence of the participant’s vaccination status from the fraction of their contact network with the same vaccination status, and then dividing by the prevalence of all other vaccination statuses. This is done if the fraction of the participant’s contact network with the same vaccination status is greater than or equal to the prevalence of their group. Conversely, if this fraction is less than the prevalence of the individual’s group, then the division is done by the prevalence of the individual’s group.

- ***Blau’s Heterogeneity Index Score.*** For each participant, we also calculated the diversity of vaccination statuses in their social network using Blau’s heterogeneity index [34], calculated as 1 minus the sum (over the numbers of doses, *k*) of the squared fraction of the participant’s contact networks with *k* doses (*p*_*k*_^*2*^*)*:
  $$H=1-\sum_{k=0}^{3}{p}_{k}^{2}.$$

Blau’s heterogeneity index scores were calculated for the number of doses (*k*=0, 1, 2, 3) for each participant’s overall contact network and dichotomized vaccination status (i.e., *k*≥1 dose vs. <1 dose) for each participant’s household, non-household, and overall contact networks.

Homophily estimates and diversity estimates were calculated across each level of vaccination, and associations with select personal and network characteristics were tested using the Kruskal–Wallis H test (for associations with categorical variables) and Spearman’s rank correlation tests (for associations with continuous variables).

#### Aim 2. Demonstration of the impact of vaccine homophily on COVID-19 transmission

To demonstrate the impact of vaccine homophily on COVID-19 transmission within and between vaccination groups under conditions of high and low vaccine efficacy, we developed a deterministic model that accounts for heterogeneity in contact patterns to assess the dynamic impact of vaccine homophily on COVID-19 transmission in BC. We analyzed the effects of vaccine homophily under two broad scenarios with low and high vaccine efficacy against infection.

##### Model assumptions

The current model was developed to demonstrate the impact of vaccine homophily in scenarios of both low and high vaccine efficacy. We stratified the model population based on the number of COVID-19 vaccine doses received. Interactions within and between groups occur at varying contact rates and preferences, reflecting how individuals interact with others based on their vaccination status. Since vaccine effectiveness against infection is not 100%, breakthrough infections can occur in all groups, with the rate depending on exposure, number of doses received, and vaccine efficacy. We assumed that newly recovered individuals have temporary protection against reinfection (denoted as ${\tau }_{j}$). If ${\tau }_{j}=0$, it implies no protection against reinfection from a previous infection; if 1, it implies perfect protection after recovery, reducing the model to the classical SIR model with sterilizing immunity after recovery. Immunity wanes at a constant rate ${\sigma }_{j}$, depending on the number of doses received. Vaccine efficacy ${\nu }_{j}$ impacts the probability of infection given contact, and we incorporated this into the force of infection ${\lambda }_{ij}$ as described below. For the unvaccinated, ${\nu }_{(j=0)}=0$, reflecting no protection from vaccination. This vaccine efficacy specifically refers to immunity against infection, as disease was not explicitly modelled, focusing instead on transmission dynamics. The recovery rate $\gamma$ is assumed to be independent of vaccination status. Without explicitly modelling importations, we assume that a constant number of susceptibles become infected due to importations at a constant rate ${f}_{j}$, which varies with vaccination status. Finally, our analysis focuses on a short period during which vaccination levels in the population remained stable.

##### Model equation

The model equations are:

$$\begin{array}{l}\frac{d{S}_{j}\left(t\right)}{dt}=-\sum\limits_{i=0}^{3}{\uplambda }_{ij}\left(t\right){S}_{j}\left(t\right)+{\sigma }_{j}{R}_{j}\left(t\right)-{f}_{j}\\ \frac{d{I}_{j}\left(t\right)}{dt}=\sum\limits_{i=0}^{3}{\lambda }_{ij}\left(t\right)\left({S}_{j}\left(t\right)+\left(1-{\tau }_{j}\right){R}_{j}\left(t\right)\right)+{f}_{j}-\gamma {I}_{j}\left(t\right)\\ \frac{d{R}_{j}\left(t\right)}{dt}=\gamma {I}_{j}\left(t\right)-\left(\left(1-{\tau }_{j}\right) \sum\limits_{i=0}^{3}{\lambda }_{ij}\left(t\right)+{\sigma }_{j}\right){R}_{j}\left(t\right),\end{array}$$

where $i,j=\mathrm{0,1},\mathrm{2,3}$ represent the number of COVID-19 doses an individual has received. Note that ${f}_{j}$ (imported infections per day) represents a constant daily rate of importation of infected vaccinated individuals into the population. Since a minimum of two doses was required for admission into BC during the study period, we assumed no importations from unvaccinated individuals or those with only a single dose into the population (${f}_{j}$ = 0 for j < 2). The non-zero values of ${f}_{j}$ are informed by a previous study [35]. The total population size is the sum of each vaccination dose group: $N={\sum }_{j=0}^{3}{N}_{j}$, where ${N}_{j}={S}_{j}+{I}_{j}+{R}_{j}$. The total population is set at 5.07 million, i.e. the population of BC.

Further details, sources, and descriptions of the variables and parameters used in the model are presented in Table 2.

**Table 2:** Descriptions of variables and parameters

**Variables and parameters**	**Description and sources**
${S}_{j}\left(t\right)$	Number of susceptible individuals
${I}_{j}\left(t\right)$	Number of infectious individuals
${R}_{j}\left(t\right)$	Number of recovered individuals
${\sigma }_{j}$	Waning rate per day for immunity against infection. Set at 1/(183 days)
${f}_{j}$	Importation rate (e.g., due to travel). Set at 150 infections per day for those with ≥ 2 doses and 0 for those with < 2 doses [35]. Assuming that travel restrictions are effective
${\tau }_{j}$	Strength of short-term protection from reinfection. At baseline: ${\tau }_{0}$=0.35,${\tau }_{1}$=0.65, ${\tau }_{2}$=0.68,${\tau }_{3}$= 0.83 (Assumed)
$\gamma$	Recovery rate per day. Set at 1/ (4 days) [36]
$\beta$	Probability of infection given contact:$0.23$ (Fitted)
${p}_{ji}$	Proportion of contacts of those with vaccination status $j$ that are of vaccination status $i$ (Estimated from survey data)
${c}_{j}$	The total number of contacts per day made by individuals with $j$ doses (Estimated from survey data)
${l}_{j}$	The level to which those with $j$ doses comply with physical distancing measures (Estimated from survey data)

##### Force of infection

The force of infection $\lambda \left(t\right)$ was defined as: (number of contacts per unit time) × (probability of disease transmission per contact) × (proportion of contacts that are infected). We used the following expression to model the force of infection:

$${\lambda }_{ij}\left(t\right)={p}_{ji}{c}_{j}\beta \left(1-{v}_{j}\right)\left(1-{l}_{j}\right)\frac{{I}_{i}}{{N}_{i}},$$

where ${\lambda }_{ij}\left(t\right)$ is the force of infection for transmitting infection from individuals with vaccination status $i$ to those with vaccination status $j$, ${p}_{ji}$ is the proportion of contacts of those with vaccination status $j$ that are of vaccination status $i$, ${c}_{j}$ is the total number of contacts per day made by individuals with $j$ doses, $\beta$ is the probability of infection given contact, ${\nu }_{j}$ is vaccine efficacy against infection for individuals with $j$ doses, ${l}_{j}$ represents the extent to which individuals with* j* doses comply with physical distancing measures. This is used to capture the heterogeneity in adherence to population-wide physical distancing measures during the study period. During this time, mask mandates and restrictions on indoor gatherings were still in place in BC. Therefore, it’s challenging to determine how respondents interpreted the survey question regarding their adherence to public health measures. One possibility is interpreting it purely as wearing a face mask or face covering during indoor contact, while another is viewing it as overall measures, including reducing contacts or avoiding large gatherings. In the former scenario, including ${l}_{j}$ in the force of infection is necessary. In the latter, the contact matrix inherently accounts for their level of adherence to public health guidelines. As a simplifying model assumption, we presumed that ${l}_{j}$ represents adherence to indoor mask-wearing during contact. In another simulation we assumed that adherence to all public health measures was already accounted for and included a supplementary figure assuming ${l}_{j}$=0 (Fig. S5 in the Supplementary Information). Under these assumptions, infections grow more rapidly, reaching higher levels. However, the direction of the force of infection essentially remains the same as when ${l}_{j}$>0. ${I}_{i}$ is the number of infected individuals who have had $i$ doses, and ${N}_{i}$ is the total number of people with $i$ doses. Parameter values were extracted from the Meta survey data.

##### Model validation

We matched model output to reported cases of COVID-19 during the survey period from February 16 to March 3, 2022. We accounted for the underreporting of cases by assuming a constant underascertainment probability (*r* = *90%*), i.e. the fraction of cases that were not detected. This is similar to the 92% reported for BC by [37], during the study period. We varied the underascertainment probability around our assumed value and found that the model’s predictions are not highly sensitive to underreporting. The model yielded a good fit to the data and provided reasonable initial conditions for subsequent model prediction. The model fit to data is shown in Fig. S2 (See Supplementary Information).

##### Model scenarios

We analyzed the impact of vaccine homophily on COVID-19 transmission dynamics under two broad scenarios. First, we assumed that vaccine efficacy in preventing infection is relatively high, representing conditions where a reasonable proportion of the population has recently received a booster vaccination. This corresponds to the situation prior to the emergence of the Omicron variant, which showed substantial escape from immunity against infection, or future scenarios where more effective vaccines are available and have been widely used. Second, we modelled a scenario with low vaccine efficacy, representing time periods where immunity has waned significantly or when the dominant variant shows low sensitivity to vaccine protection. We further considered each of the above scenarios with and without homophily. For the former, we used contact-related parameter (${p}_{ji},{c}_{j}{l}_{j}$) values estimated from the survey data, combining both household and non-household contacts, while in the latter, we calculated a weighted average for each of the parameters to eliminate the impact of vaccine homophily. That is, for the without homophily scenario, the total number of contacts (${c}_{j}$) for each vaccination group and the proportion of contacts individuals make with those in their group and everyone else, as well as the level of adherence to physical distancing measures, are the same for each group regardless of vaccination status.

Below, we describe the parameter values we used for model simulations under various scenarios.

The values of the invariant parameters are presented in Table 2. With vaccine homophily, we used the following parameters as estimated from the survey data: Proportion of contacts of those with vaccination status $j$ that are of vaccination status $i$ (${p}_{ji}$).

${p}_{00}=0.45,{p}_{01}=0.02,{p}_{02}=0.39,{p}_{03}=0.14{, p}_{10}=0.17,{p}_{11}=0.08,{p}_{12}=0.61,{p}_{13}=0.14, {p}_{20}=0.11,{p}_{21}=0.02,{p}_{22}=0.69,{p}_{23}=0.18,{p}_{30}=0.03,{p}_{31}=0.01,{p}_{32}=0.32, {p}_{33}=0.64$. For the total number of contacts per day made by individuals with $j$ doses (${c}_{j}$*)*, we estimated values from the survey data as follows. ${c}_{0}=2.11,{c}_{1}= 2.68,{c}_{2}=2.19,{c}_{3}=1.73$, per day. The level of compliance to physical distancing measures (${l}_{i}$) is calculated from the survey data and obtained as follows: ${l}_{0}=0.134, {l}_{1}=0.174,{l}_{2}=0.349,{l}_{3}=0.817$. Furthermore, to generate a scenario of conditions without vaccine homophily, we eliminate the impact of vaccine status homophily in the number of contact per day by finding the average weighted contact ${c^\prime}_{j}$ per day for the population as follows: $c^\prime={c}_{1}{p}_{1}+{c}_{2}{p}_{2}+{c}_{3}{p}_{3}=2.43$ per day where ${p}_{j}$ is the prevalence of each vaccination status group which was calculated from vaccination coverage data as 0.07, 0.03, 0.42, and 0.48 for ${p}_{0}$, ${p}_{1}$, ${p}_{2}$, and ${p}_{3}$ respectively [33]. A similar procedure was used to calculate the weighted average rate of adherence to public health measures, $l^\prime$=*0.514*.

For the no-homophily scenario, we set ${p^\prime}_{ji}$ (Proportion of contacts of those with vaccination status $j$ that are of vaccination status $i$) as the proportion of individuals with $i$ doses in the population, for all $j$. Under conditions of low vaccine efficacy, we used: ${v}_{0}$= *0,*${v}_{1}$= *0.001,*${v}_{2}$= *0.02,*${v}_{3}$= *0.07,*${\tau }_{0}$= *0.20,*${\tau }_{1}$= *0.40,*${\tau }_{2}$= *0.65,*${\tau }_{3}$= *0.80*.

Under conditions of high vaccine efficacy and higher temporary protection after recovery, we used: ${v}_{0}$= *0,*${v}_{1}$= *0.60,*${v}_{2}$= *0.89,*${v}_{3}$= *0.93,*${\tau }_{0}$= *0.80,*${\tau }_{1}$= *0.85,*${\tau }_{2}$= *0.90,*${\tau }_{3}$= *0.97*.

For these four scenarios, we assumed $\beta$=0.6, which is higher than the estimated value in Table 2, to generate scenarios that would allow for an increase in the number of infections. The values of $\tau$ and $v$ are also assumed to represent high and low vaccine efficacy scenarios.

### Ethics review

The study protocol was approved by the Research Ethics Board of Simon Fraser University (Protocol #30000753). All participants provided informed consent before completing the survey,

## Results

### Aim 1. Characterization of vaccine homophily and its relationship to COVID-19 transmission dynamics

Facebook and Instagram advertisements were displayed to 266,894 users. A total of 3659 participants initiated the survey and provided informed consent to participate in the study. After exclusion of responses that were of poor quality or had missing data, the final analytical sample size was 1185.

The unweighted sample was disproportionately White (86.9%), female (58.1%), had higher income (≥ $90,000, 58.7%), and had been born in Canada (82.8%) (Table 3). Statistical weights were used to align these factors with the population distribution based on the 2016 Canadian Census Profile for British Columbia, Canada.

**Table 3:** Characteristics of the study population, weighted for BC population characteristics based on 2016 census profile

**Variable**	**Weighted**
**Age**, mean (SD)	47.49 (17.63)
**Gender**, *n* (%)	
Male	563.9 (47.6)
Female	590.3 (49.8)
Non-binary	30.5 (2.6)
**Ethnicity**, *n* (%)^a^	
White	761.2 (64.2)
Asian	175.7 (14.8)
Indigenous	173.9 (14.7)
Other	73.9 (6.2)
**Education level**, *n* (%)	
Some high school	112.1 (9.5)
High school diploma or equivalent	418.9 35.4)
College or trades certificate or diploma	257.8 (21.8)
Some university	107.8 (9.1)
University degree or higher (e.g., Bachelors, Masters, PhD, JD, MD)	288.1 (24.3)
**Household income**, *n* (%)	
< $30,000	227.3 (19.2)
$30,000–$59,999	287.5 (24.3)
$60,000–$89,999	231.9 (19.6)
≥ $90,000	438.0 (37.0)
**Born in Canada**, *n* (%)	813.3 (69.8)
**Health authority**, *n* (%)	
Vancouver Coastal	310.2 (26.2)
Fraser	419.3 (35.4)
Interior	192.8 (16.3)
Island	189.2 (16.0)
Northern	73.2 (6.2)

^a^Due to the small sample sizes in most ethnicity categories, the statistical weight for the ethnicity variable was generated based on a binary variable measuring whether participants were either White or another ethnicity. While suboptimal, this was necessary to achieve convergence of the raking estimation algorithm. Weighted estimates may not round to whole numbers or sum to 100%

Table 4 presents additional descriptive statistics about self-reported COVID-19 diagnosis history and self-reported compliance with provincial COVID-19 prevention guidelines, stratified by self-reported vaccination status. Statistical comparisons across these variables indicate that participants who had received more doses of the COVID-19 vaccine were less likely to report a previous COVID-19 diagnosis (*p* < 0.0001) and were more likely to report higher compliance with provincial COVID-19 prevention guidelines (*p* < 0.0001).

**Table 4:** Personal indicators of COVID-19 risk, weighted

	0 Doses *n* = 234.4	1 Dose *n* = 20.6	2 Doses *n* = 243.9	≥ 3 Doses *n* = 685.7
**COVID-19 Diagnosis/Infection**, *n* (%)				
No, and I do not think I have had COVID-19	76.3 (32.0)	10.9 (59.6)	73.2 (30.4)	486.2 (74.0)
No, but I think I have had COVID-19. I just never received a test and/or diagnosis.	86.6 (37.4)	2.8 (15.1)	92.0 (38.1)	112.9 (17.2)
Yes, I have been diagnosed with COVID-19	69.0 (29.8)	4.7 (25.3)	75.9 (31.5)	57.9 (8.8)
**Compliance with COVID-19 Guidelines**, *n* (%)				
Not At All	28.8 (12.3)	1.0 (5.0)	14.6 (6.0)	0.4 (0.1)
Not Very Closely	77.6 (33.1)	4.5 (22.0)	59.0 (24.3)	13.3 (2.1)
Somewhat Closely	97.1 (41.4)	9.9 (48.3)	94.6 (39.0)	116.6 (18.0)
Very Closely	30.9 (13.2)	5.1 (24.8)	74.4 (30.7)	515.9 (79.8)

Values may not round to whole numbers or sum to 100% due to missing observations on some variables and statistical weighting

Table 5 presents descriptive statistics for participant-reported descriptions of their household and non-household contacts stratified according to self-reported vaccination status. Statistical comparisons across these variables indicate that participants who had received more doses of the COVID-19 vaccine had networks with higher average numbers of doses (*p* < 0.0001) and had a greater proportion of network contacts with at least one vaccine dose (*p* < 0.0001). With regard to network vaccine heterogeneity, participants with fewer vaccine doses had more heterogeneous networks according to Blau’s heterogeneity index (*p* < 0.0001).

**Table 5:** Social network indicators of COVID-19 risk

	0 Doses *n* = 234.4	1 Dose *n* = 20.6	2 Doses *n* = 243.9	≥ 3 Doses *n* = 685.7
Proportion of overall contacts with prior COVID-19, *n* (%)				
A few of them (i.e., 0–20%)	94.5 (40.6)	16.6 (80.6)	100.6 (41.2)	493.5(72.0)
Some of them (i.e., 21–40%)	34.7 (14.9)	0.1 (0.4)	39.6 (16.2)	96.7 (14.1)
Around half of them (i.e., 41–60%)	25.0 (10.7)	0.0 (0.1)	49.1 (20.1)	57.2 (8.3)
Most of them (i.e., 61–80%)	54.2 (23.3)	2.8 (13.7)	34.6 (14.2)	33.9 (4.9)
Nearly all of them (i.e., 80–100%)	24.5 (10.5)	1.1 (5.2)	20.1 (8.2)	4.4 (0.6)
Proportion of overall contacts adhering closely to guidelines, *n* (%)				
A few of them (i.e., 0–20%)	31.4 (13.4)	3.9 (18.8)	29.9 (12.3)	30.7 (4.5)
Some of them (i.e., 21–40%)	41.8 (17.8)	3.9 (18.8)	46.9 (19.2)	35.8 (5.2)
Around half of them (i.e., 41–60%)	45.2 (19.3)	5.4 (26.4)	39.9 (16.4)	79.2 (11.6)
Most of them (i.e., 61–80%)	93.4 (39.9)	7.4 (35.9)	78.5 (32.2)	271.3 (39.6)
Nearly all of them (i.e., 80–100%)	22.6 (9.7)	0.0 (0.1)	48.5 (19.9)	268.6 (39.2)
Proportion of overall contacts vaccinated, *n* (%)				
A few of them (i.e., 0–20%)	20.0 (8.5)	2.3 (10.9)	12.8 (5.3)	10.3 (1.5)
Some of them (i.e., 21–40%)	40.8 (17.4)	0.3 (1.7)	14.2 (5.8)	6.0 (0.9)
Around half of them (i.e., 41–60%)	68.4 (29.2)	1.7 (8.4)	42.5 (17.4)	9.3 (1.4)
Most of them (i.e., 61–80%)	68.1 (29.0)	3.8 (18.5)	88.2 (36.2)	128.6 (18.9)
Nearly all of them (i.e., 80–100%)	37.2 (15.9)	12.5 (60.5)	86.2 (35.4)	526.2 (77.3)
Number of non-household contacts, mean (SD)	21.76 (19.24)	13.30 (12.86)	20.29 (16.03)	15.52 (16.38)
Number of non-household contacts with known vaccine status, mean (SD)	10.21 (9.75)	10.40 (10.50)	10.67 (9.82)	10.97 (12.17)
Vaccination status of non-household contacts, mean (SD)				
0 doses	3.73 (6.49)	1.23 (3.23)	1.13 (3.53)	0.35 (1.21)
1 dose	0.17 (0.55)	1.03 (1.91)	0.19 (0.66)	0.18 (0.97)
2 doses	4.41 (6.54)	4.65 (6.37)	7.19 (9.05)	4.34 (9.42)
3 doses	1.90 (4.66)	3.49 (5.07)	2.15 (3.46)	6.10 (8.01)
Household size, mean (SD)	1.85 (1.42)	1.90 (0.79)	2.14 (1.28)	1.76 (1.02)
Vaccination status of household contacts, mean (SD)				
Unknown	0.11 (0.46)	0.06 (0.35)	0.06 (0.31)	0.00 (0.00)
0 doses	1.09 (1.38)	0.56 (1.07)	0.29 (0.92)	0.03 (0.17)
1 dose	0.05 (0.22)	0.40 (0.81)	0.03 (0.18)	0.01 (0.12)
2 doses	0.40 (0.83)	0.32 (0.48)	1.38 (1.11)	0.26 (0.59)
3 doses	0.21 (0.52)	0.56 (0.91)	0.38 (0.60)	1.46 (0.97)
Calculated measures				
Average number of doses among overall contacts, mean (SD)	1.31 (0.79)	1.81 (0.70)	2.00 (0.49)	2.61 (0.45)
Average number of doses among household contacts, mean (SD)	0.83 (1.06)	1.59 (1.11)	1.93 (0.75)	2.81 (0.43)
Average number of doses among non-household contacts, mean (SD)	1.47 (0.89)	1.88 (0.57)	2.02 (0.56)	2.55 (0.55)
% of overall contacts with at least 1 dose, mean (SD)	0.58 (0.31)	0.85 (0.22)	0.89 (0.18)	0.97 (0.12)
% of household contacts with at least 1 dose, mean (SD)	0.36 (0.45)	0.74 (0.41)	0.86 (0.30)	0.99 (0.10)
% of non-household contacts with at least 1 dose, mean (SD)	0.65 (0.36)	0.91 (0.16)	0.90 (0.20)	0.96 (0.15)
Vaccine Homophily, mean, (SD)	0.27 (0.60)	-0.34 (0.63)	0.35 (0.57)	0.42 (0.60)
Network heterogeneity for ≥ 1 dose, mean (SD)	0.31 (0.20)	0.17 (0.18)	0.13 (0.19)	0.04 (0.10)
Household heterogeneity for ≥ 1 dose, mean (SD)	0.11 (0.27)	0.07 (0.20)	0.06 (0.17)	0.01 (0.06)
Non-household heterogeneity for ≥ 1 dose, mean (SD)	0.20 (0.20)	0.11 (0.18)	0.10 (0.17)	0.03 (0.10)
Network heterogeneity, same number of doses in overall contacts, mean (SD)	0.41 (0.20)	0.42 (0.21)	0.33 (0.21)	0.25 (0.22)
Network heterogeneity, same number of doses in household contacts, mean (SD)	0.13 (0.28)	0.08 (0.21)	0.15 (0.24)	0.06 (0.17)
Network heterogeneity, same number of doses in non-household contacts, mean (SD)	0.30 (0.23)	0.34 (0.24)	0.24 (0.22)	0.22 (0.22)

Values may not round to whole numbers or sum to 100% due to missing observations on some variables and statistical weighting

**Fig. 2:**
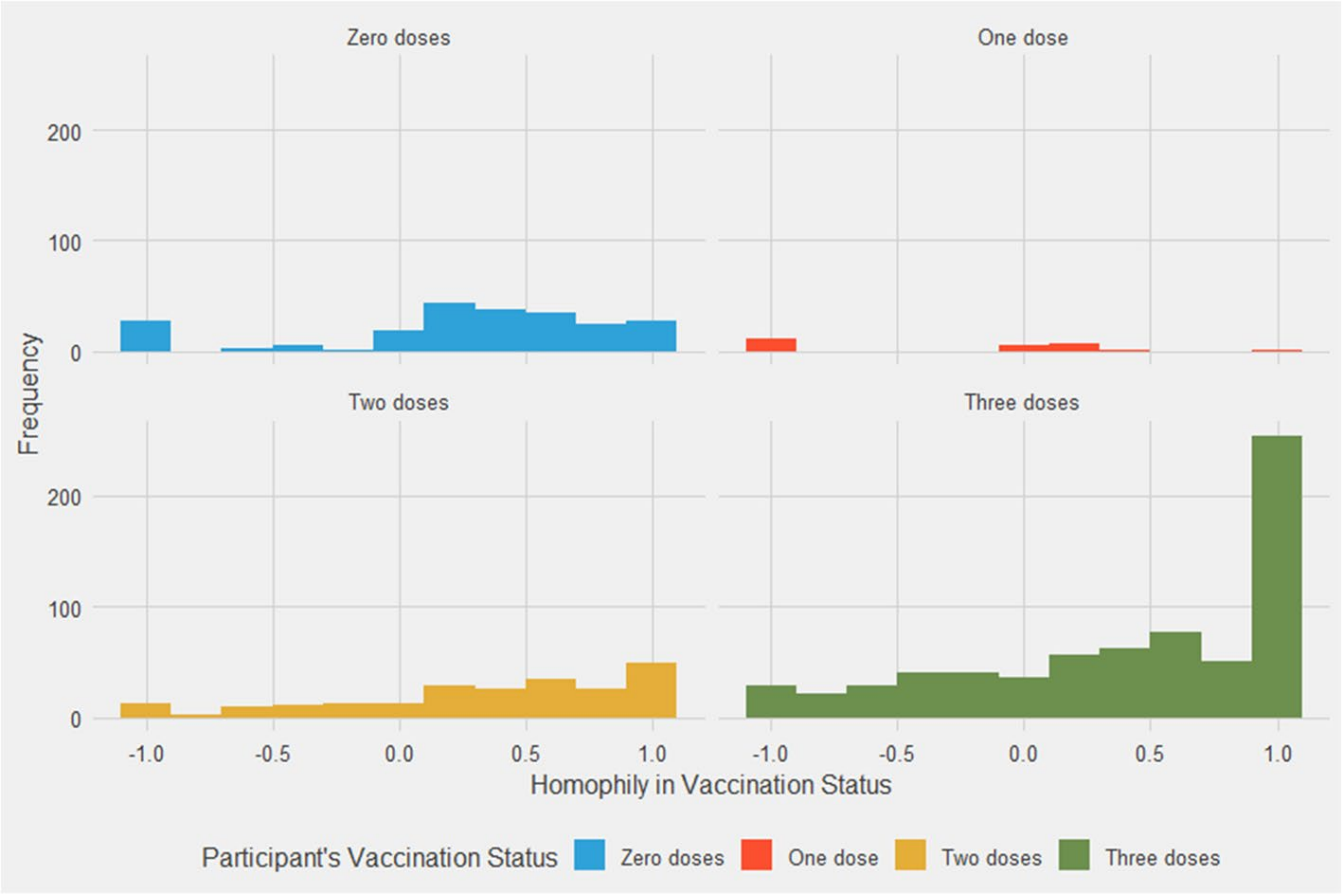
Distribution of vaccine homophily scores, by participant vaccination status

Figure 2 plots the distribution of the vaccine homophily scores, ranging from -1.0 to 1.0, for individuals with 0, 1, 2, or 3 vaccine doses. Kruskal-Wallis test indicates significant differences in homophily between individuals with differing numbers of vaccine doses (*p* < 0.0001). Refer to Fig. S1 in the Supplementary Information for an alternative version of Fig. 2, which displays homophily scores with a customized vertical axis.

**Fig. 3:**
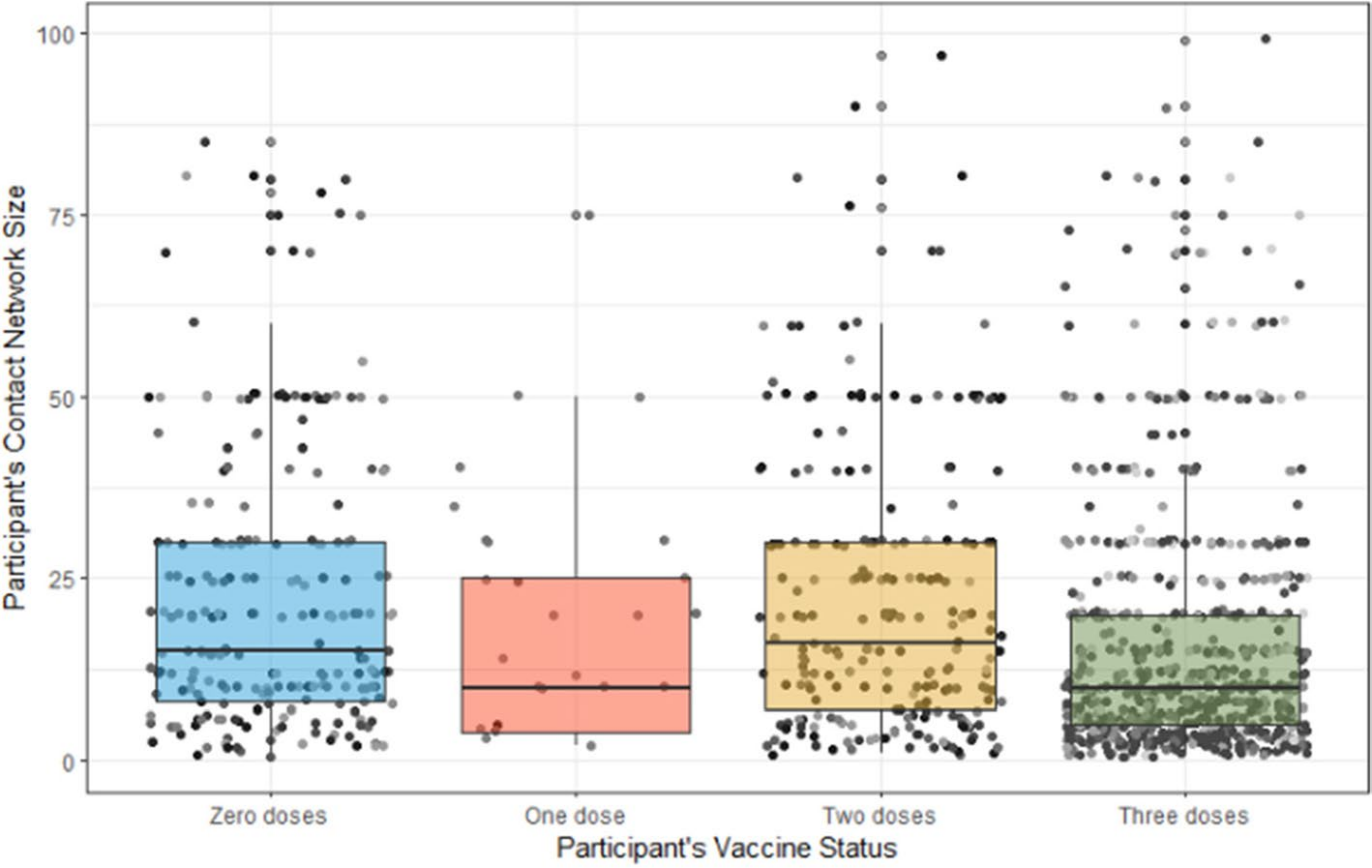
Homophily and Contact Network Size by Quantile. Figure Note: Each box in the boxplot spans from the first quartile (Q1) to the third quartile (Q3) of the data, representing the middle 50% of the data points for each vaccine status category. The black horizontal bar within each box represents the median of the data. In the context of this boxplot, it indicates the median network size for participants within each vaccine status category. The vertical lines, known as whiskers, extend from the upper and lower edges of the box to the highest and lowest values within a specific range. This range is typically defined as 1.5 times the interquartile range (IQR) above the upper quartile and below the lower quartile. Values outside this range are considered outliers and are not included in the whiskers. In this plot, the whiskers represent the spread of the network size data points, excluding outliers, for each vaccine status category

Figure 3 shows boxplots of the participants’ contact network sizes stratified according to vaccination status. Participants with more vaccine doses—particularly those with three or more doses—tended to have smaller average network sizes (Spearman’s *r* =  − 0.217, *p* < 0.0001). Pairwise comparisons indicated that those who received zero doses exhibited a significantly different network size compared to those who received three doses, *p* < 0.0001. However, no significant difference was found between individuals who received zero doses and those who received either one dose (*p* = 0.958) or two doses (*p* = 0.958). Furthermore, individuals who received three doses showed a significantly different network size compared to those who received one dose (*p* = 0.031) and two doses (*p* < 0.0001). There was no significant difference between individuals who received one dose and those who received two doses (*p* = 0.958).

Finally, we also tested the association between vaccine homophily and network size, finding that higher network size was associated with lower vaccine homophily (Spearman’s *r* = -0.114, *p* < 0.0001).

### Aim 2. Demonstration of the impact of vaccine homophily on COVID-19 transmission

**Fig. 4:**
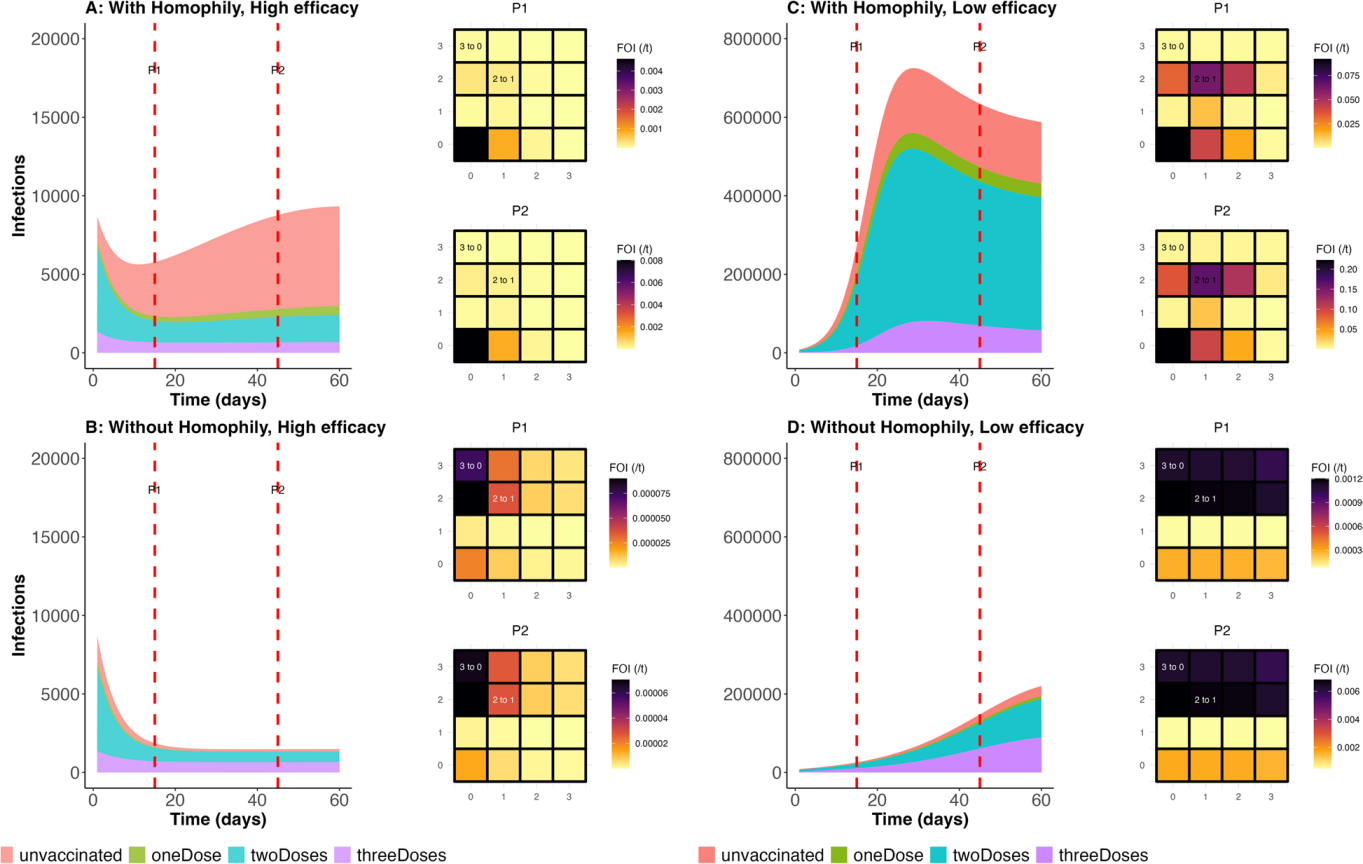
Number of Infections and Force of Infection: Assessment of the Impact of Homophily Under Scenarios of Low and High Vaccine Efficacy. **A** Number of infections under a scenario with vaccine homophily and high vaccine efficacy. The trajectory is colour-coded by vaccination status. Heat maps P1 and P2 show the force of infection at 15 and 45 days, respectively. **B** Number of infections under a scenario without vaccine homophily and with high vaccine efficacy. **C** The number of infections per day for various vaccination groups under a scenario with vaccine homophily and low vaccine efficacy. **D** Daily number of infections according to vaccination status under a scenario without homophily and low vaccine efficacy. The following parameter values were used under conditions with vaccine homophily. The horizontal and vertical axes on the heat maps represent vaccination status. The numbers within the heat maps indicate which group is transmitting infection to which other group: “2 to 1” indicates that individuals with 2 doses transmit to those with only 1 dose on that grid, and “3 to 0” indicates that those with 3 doses transmit to unvaccinated individuals on that grid

Our deterministic mathematical model tested the impact of vaccine homophily on COVID-19 transmission dynamics under conditions of high and low vaccine efficacy. To illustrate these effects, Fig. 4 presents four scenarios describing the intersection of vaccine homophily and vaccine efficacy. Each panel in the figure shows the number of infections from 0 to 60 days and two heat maps characterizing the force of infection at 15 (P1) and 45 (P2) days. Overall, in both low and high vaccine efficacy scenarios, the presence of vaccine homophily contributed to higher levels of epidemic growth. We describe each of the four scenarios in the following section to highlight the interaction between homophily and vaccine efficacy. The model’s initial conditions were established to reflect the vaccination uptake levels in British Columbia (BC) as of February 16, 2023. It was then fitted to case report data accumulated during the study period. After calibration, the final values of the state variables in the SIR model (Susceptible, Infected, Recovered) were used to initialize the model for each simulated scenario. Subsequently, the model was simulated for two scenarios, each spanning a 60-day period.

#### With vaccine homophily and high vaccine efficacy (Fig. 4A)

In this scenario, the epidemic is primarily driven and sustained by unvaccinated individuals, as indicated by the darker heat maps. While infections increase slowly in the vaccinated groups, epidemic growth is rapid among the unvaccinated. The force of infection in this scenario reveals that transmission is predominantly sustained within the unvaccinated group. There is only a slight impact on the one-dose group due to suboptimal immunity. However, there is minimal effect on the two-dose and three-dose groups, owing to the high vaccine efficacy.

#### Without vaccine homophily and high vaccine efficacy (Fig. 4B)

In this scenario, the unvaccinated are mixing randomly with the vaccinated, and the epidemic quickly stabilizes as the unvaccinated obtain secondary benefits from the predominantly fully vaccinated population. Meanwhile, disease importation sustains transmission at a steady state. Furthermore, a substantial number of infections in the unvaccinated group are caused by the vaccinated groups, because contacts are primarily driven by group sizes (see Figure S3 in the Supplementary Information, which describes contact between groups). On the other hand, high vaccine efficacy against infection limits the force of infection from the unvaccinated group to the optimally vaccinated group.

#### With vaccine homophily and low vaccine efficacy (Fig. 4C)

In this scenario, due to the low vaccine efficacy, major outbreaks occur among the unvaccinated and suboptimally vaccinated, while infections quickly stabilize among the fully vaccinated, despite the low vaccine efficacy. The epidemic is primarily driven by the unvaccinated and those with two doses. This could be due to the large size of the two-dose group combined with the relatively low vaccine efficacy. Although each individual has partial protection, the overall population size and low vaccine efficacy result in a substantial force of infection within the two-dose group. Unvaccinated individuals significantly impact those within their group and have some impact on those with one or two doses, but minimal impact on the group with three or more doses, as the mixing pattern limits intergroup contact. A similar pattern is observed in the group with two doses.

#### Without vaccine homophily and with low vaccine efficacy (Fig. 4D)

In this scenario, each group affects both itself and other groups equally, although the strength of the impact depends on the vaccination status and the size of the group. Moreover, the fully vaccinated group has a substantial impact on both the unvaccinated and partially vaccinated, as contact is driven by group sizes, coupled with the low vaccine efficacy.

#### Without vaccine homophily and with vaccine efficacy for Omicron

In this scenario (See Supplementary Information Figure S4), published vaccine efficacy values against infection with the Omicron variant were used [38]. The two-dose group drives infections in both the one-dose and unvaccinated groups, albeit with a somewhat reduced impact on those with three or more doses. Furthermore, the influence of the unvaccinated group on the two- and three-dose groups is less pronounced than the converse. These dynamics likely stem from the relatively low vaccine efficacy against the Omicron variants and the absence of vaccine status homophily, which allows the prevalence of each vaccination group to drive contact patterns. This scenario was designed to reflect the situation in British Columbia where homophily-enhancing measures, such as vaccination passports, were relaxed shortly after the study period. This may have led to a reduction in homophily. Given that the Omicron variant was the dominant strain in BC at the time, we focused on vaccine efficacy against the Omicron variant.

### Interpretation

#### Primary findings

This study was performed to characterize observable patterns of vaccine homophily and examine the impact of vaccine homophily on COVID-19 transmission both within and between vaccination status groups under conditions of high and low vaccine efficacy. The results indicated the occurrence of vaccine homophily, with a large proportion of the participants’ network contacts having the same number of vaccine doses as the participants themselves. Even adjusting for the population prevalence of each vaccine dose, those with zero, two, or three doses reported greater than expected levels of homophily. Similarly, the average number of doses received by household and non-household contacts was highest among those with ≥ 3 doses and lowest among those with 0 doses, demonstrating a higher prevalence of vaccination within the networks of vaccinated individuals relative to unvaccinated individuals. Those who were unvaccinated also had more diverse social networks with regard to vaccine status, were more likely to report previous COVID-19 infection and had larger social network sizes. Mathematical models demonstrated that these dynamics contribute to elevated transmission overall under conditions of high vaccine efficacy, and transmission is driven primarily by unvaccinated individuals infecting other unvaccinated individuals. Under conditions of low vaccine efficacy, within-group transmission among unvaccinated individuals remains high, but there is also considerable impact of unvaccinated transmission on suboptimally vaccinated individuals. Those with suboptimal protection (e.g., two doses) also experience considerable within-group transmission due to high contact rates with other suboptimally protected contacts within their network.

One factor contributing to these patterns is a higher level of observed vaccine homophily among household contacts compared to non-household contacts. Indeed, among unvaccinated participants, only 39% of household contacts had one or more doses of the COVID-19 vaccine, compared to 68% of non-household contacts. We also found that vaccine homophily appears to decrease as social network size increases, suggesting that smaller networks are more similar to one another than larger networks. This is consistent with the empirical expectation that people tend to associate with people like themselves and are more different from those who are more socially distant [11].

To our knowledge, there have been few reports of empirically measured COVID-19 vaccine homophily. However, our findings that vaccine homophily has important implications for understanding the transmission of COVID-19 were consistent with previous modelling studies [12, 13, 15]. In situating our findings within these previous studies, it is important to note that the impact of vaccine homophily differs according to the level of vaccine efficacy. Under conditions of high vaccine efficacy, transmission occurs largely among unvaccinated individuals. Meanwhile, contact patterns put suboptimally vaccinated individuals at risk of infection under conditions of low vaccine efficacy. In contrast, fully vaccinated individuals experience a lower risk of infection. Furthermore, contrary to some narratives that blame unvaccinated individuals for driving the epidemic under conditions of low vaccine efficacy, we found that the force of infection is substantially influenced by group sizes in ‘without homophily’ scenarios. Additionally, a sizeable portion of the force of infection among unvaccinated individuals originates from outside their group, a trend that becomes more pronounced when vaccine efficacy is low. With vaccine homophily, unvaccinated individuals pose significantly greater risk to other unvaccinated individuals than to other groups. The impact of unvaccinated individuals on fully vaccinated individuals is considerable only when there is low vaccine homophily, and vaccine efficacy is low. For all the scenarios we considered, the impact of homophily is amplified by increased probability of infection per contact.

The overrepresentation of the unvaccinated in the total number of infections (Fig. 4A) is similar to findings from Canada, based on case-level vaccine history data. Among individuals aged 5 years and older, the unvaccinated constituted approximately 30% of the total reported cases since the onset of the vaccination rollout, as of June 10, 2022. As the vaccination rollout progresses, the limited testing capacity has resulted in the targeted testing of the high-risk population for severe disease, which coincides with the group prioritized during the vaccination rollout. Consequently, this bias in the case report data indicates that reported case data by vaccination status may not accurately reflect the distribution of infections by vaccination status at the population level [33]. For example, in BC, the unadjusted data indicated that the unvaccinated accounted for 14.2% of the total cases, whereas the age-adjusted cases per 100,000 population in the province showed that unvaccinated groups accounted for 58% in March 2022 [39]. This finding is consistent with the initial conditions of our model at the beginning of March 2022.

Taken together, our findings are worrisome, particularly when considering the risk for transmission within households, which are known to account for a significant proportion of COVID-19 infections [40–42]. Furthermore, the high risk of infection among unvaccinated individuals, even with an effective vaccine available, underscores the need for vaccine-status-specific COVID-19 prevention measures. These measures are crucial as unvaccinated individuals can significantly contribute to hospitalizations, even when they are a minority [43], which might raise important questions about health equity. Such measures may include mask mandates, physical distancing rules, and proof of vaccination requirements. Given the group transmission dynamics that arise due to household and non-household contact networks, it is important to engage these populations to address vaccine hesitancy [44–50]. This will likely require community-based and culturally aware public health interventions that can help reduce vaccine hesitancy. Indeed, rather than viewing unvaccinated individuals as a threat to public health, it should be taken as an opportunity to educate and work with these individuals to address their concerns, particularly given the skepticism that may be associated with the emergency use authorizations that have allowed the rapid rollout of COVID-19 vaccines [46, 47].

### Limitations

This study had some limitations that should be taken into consideration when interpreting our findings. First, we note that our findings are relevant to the promotion of vaccines across the population and emphasize the importance of continued vaccine research and efforts to provide ongoing protection as vaccine-induced immunity wanes. However, our data are from a period in which individuals were receiving third doses and facing the rising prevalence of the Omicron variant. Therefore, our results should not be read as predictive scenarios. Rather, they should be interpreted in the context of a pandemic-related mass-vaccination effort, during which there was uneven uptake of vaccines across social networks due to a variety of factors within and outside the control of individuals.

Second, we note that our survey utilized an online, opt-in convenience sampling methodology to study the effects of interest. Online sampling is now a widespread methodology, particularly since the decline in reliability of other opt-in sampling strategies such as random digit dialing methods. Point estimates from this study are therefore likely to be non-representative and may be biased. However, we note that studies show that epidemiological and behavioural estimates from web and telephone surveys are typically comparable, and that online samples may have advantages to other survey methods (e.g., reduced favourable reporting; [51]). This is because the direction of bias may be random. While population weights may partially adjust for this issue, the direction and magnitude of potential biases are unknown. Replication in a population-based sample is warranted. Furthermore, our weighting method does not make any assumptions regarding the statistical relationship between weighting variables (i.e., each variable included in the weighting variable is iteratively fit until target weights are met, without trying to match marginal weights).

Third, it is important to acknowledge that our sample size was relatively modest. Replicating our findings in a larger sample could offer more robust evidence and enhance the accuracy of our measurements. However, we must acknowledge that replicating the study will present significant challenges, particularly given the current stage of the pandemic. Tracking the vaccination statuses of individuals within contact networks may prove to be a daunting task.

Fourth, self-reported data may be unreliable, particularly estimates regarding characteristics of participants’ social networks. People may be overly confident in estimating their network’s vaccination status, guideline compliance, and vaccine history of their social network contacts, which may result in a systematic bias toward the hypothesis that vaccine status homophily exists. Fifth, we do not intend to imply causality in describing any of the relationships between vaccine status and vaccine homophily. Further qualitative and quantitative studies are needed to understand the processes that give rise to vaccine homophily and how best to respond to these network characteristics.

Our modelling assumptions did not consider vaccine efficacy against infectiousness, except that if infection itself is prevented, so is infectiousness. We made this choice partly because the evidence for a reduction in infectiousness due to vaccination is still emerging [52, 53]. Additionally, during the study period, almost everyone in the population had been exposed to the infection, which suggests that the majority might exhibit some reduction in infectiousness due to vaccination or natural immunity. This generally impacts the probability of infection given a contact. A recent study suggests a 22% reduction in infectiousness post-vaccination [52]. We found that our results remain robust with a reduction in infectiousness around that value, which slightly impacts the total infection. Detailed modelling of the interaction between acquired and natural immunities, and their efficacy against infectiousness, would require more data and details, which are beyond the scope of the current study.

## Conclusion

The present study identified evidence of homophily in COVID-19 vaccine uptake. Unvaccinated individuals are more likely to have unvaccinated network contacts, conditions that create increased risk of COVID-19 transmission among unvaccinated individuals. Nevertheless, vaccine homophily varies considerably, and further research is needed to understand the factors that shape vaccine homophily within social networks. Vaccine status-specific prevention guidelines may help to mitigate the risks to communities posed by the unique risk profiles of unvaccinated individuals.

### Supplementary Information

**Additional file 1.**

## Data Availability

Data sets generated during the current study are available from Dr. Kiffer Card.
