# Supplementary material for: The role of vaccine status homophily in the COVID-19 pandemic: a cross-sectional survey with modelling
Source: BMC Public Health. 2024 Feb 14;24:472. doi: 10.1186/s12889-024-17957-5 (PMC10868109; doi:10.1186/s12889-024-17957-5)
Supplement: Supplementary file 1 — Additional file 1. [file 12889_2024_17957_MOESM1_ESM.docx]

# **Supplementary Information**

The Supplementary Information contains additional figures and tables. Each figure and table is referenced in the main text.


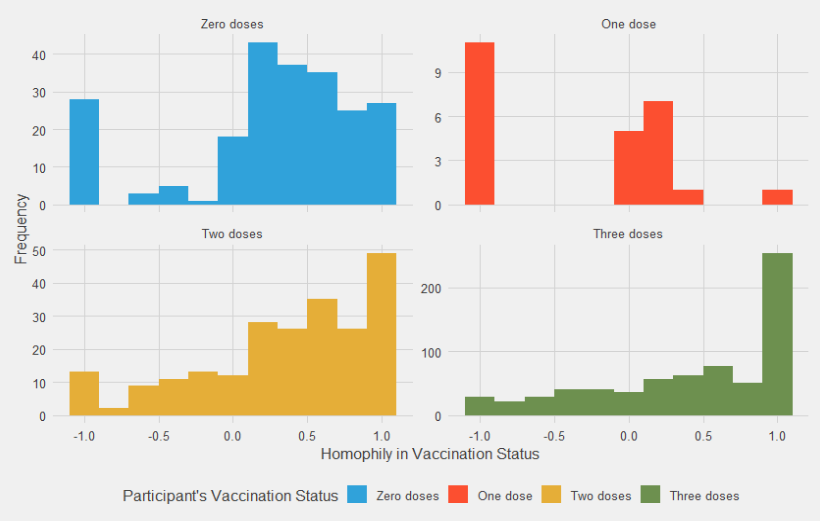


**Figure S1.** Distribution of Vaccine Homophily Scores, by Participant Vaccination Status


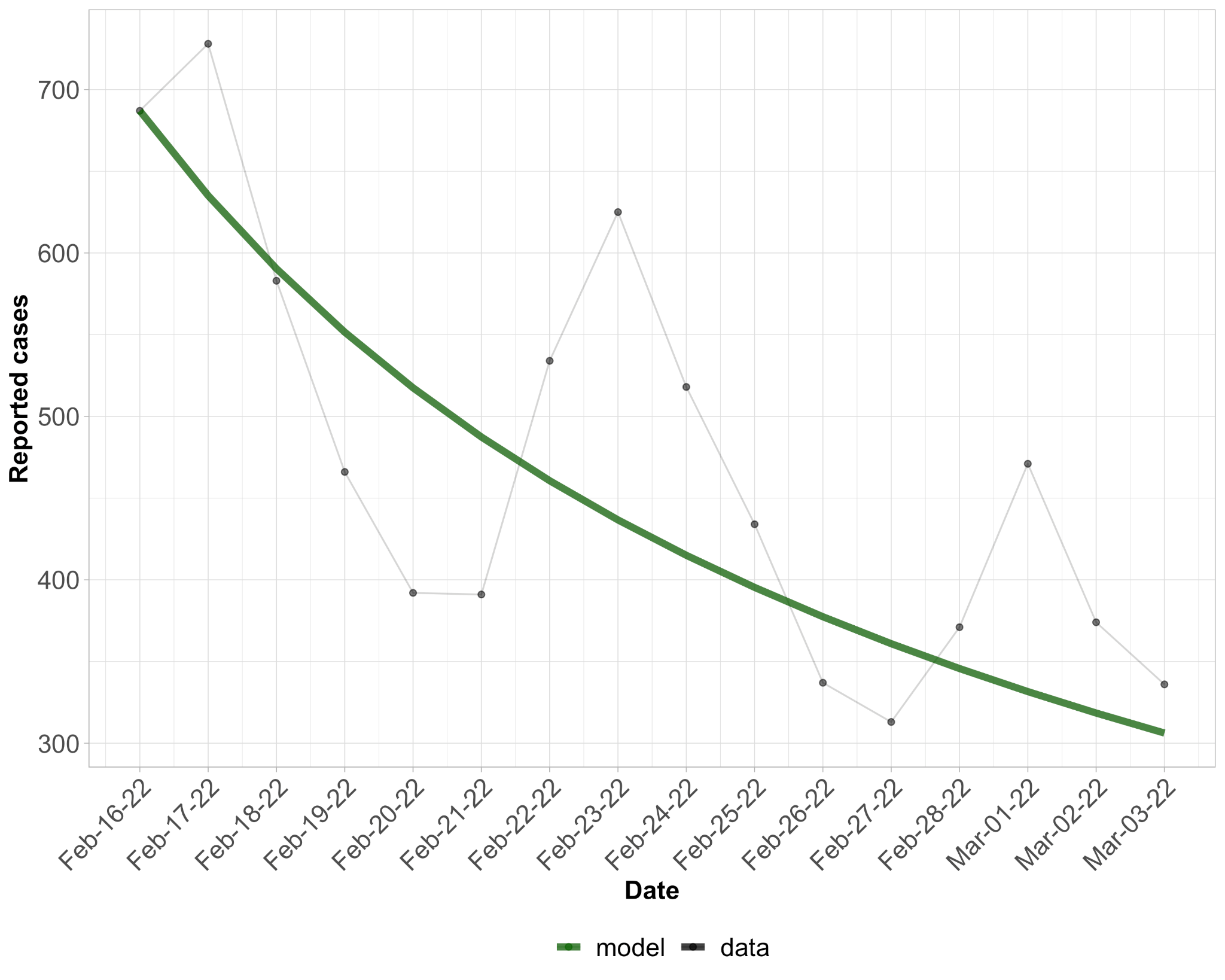


**Figure S2.** Model Fit to Reported Cases From February 16, to March 3, 2022.

The green line indicates the model output, while the gray connected dots show reported cases during the study period.


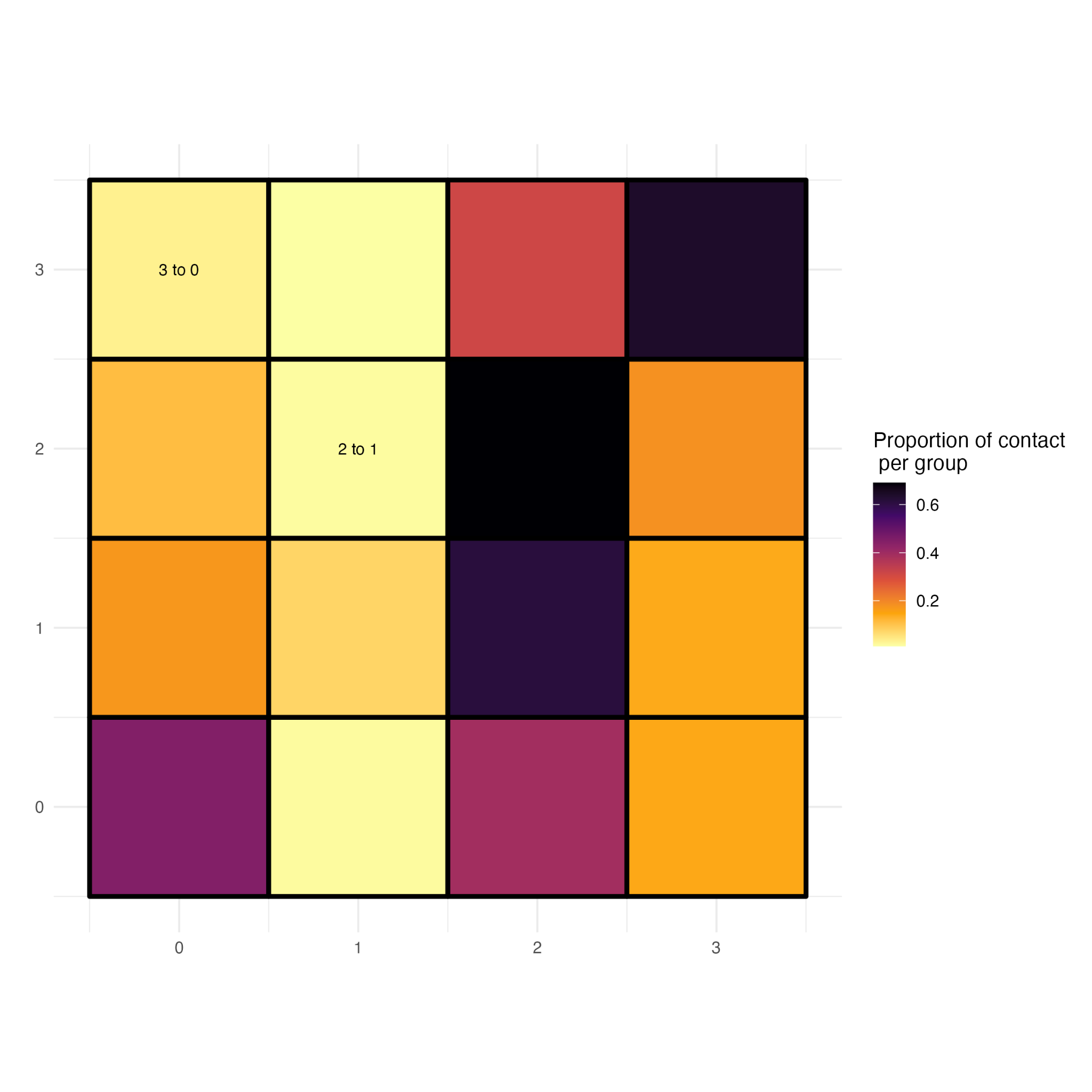


**Figure S3.** A Null Model Showing the Proportions of Contacts of Each Vaccination Group.

Each panel shows the proportion of contacts either within or outside their vaccination group. “2 to 1” indicates the proportion of contacts of individuals in the 2-dose group that have had 1 dose of vaccine. Axis labels indicate vaccination status.


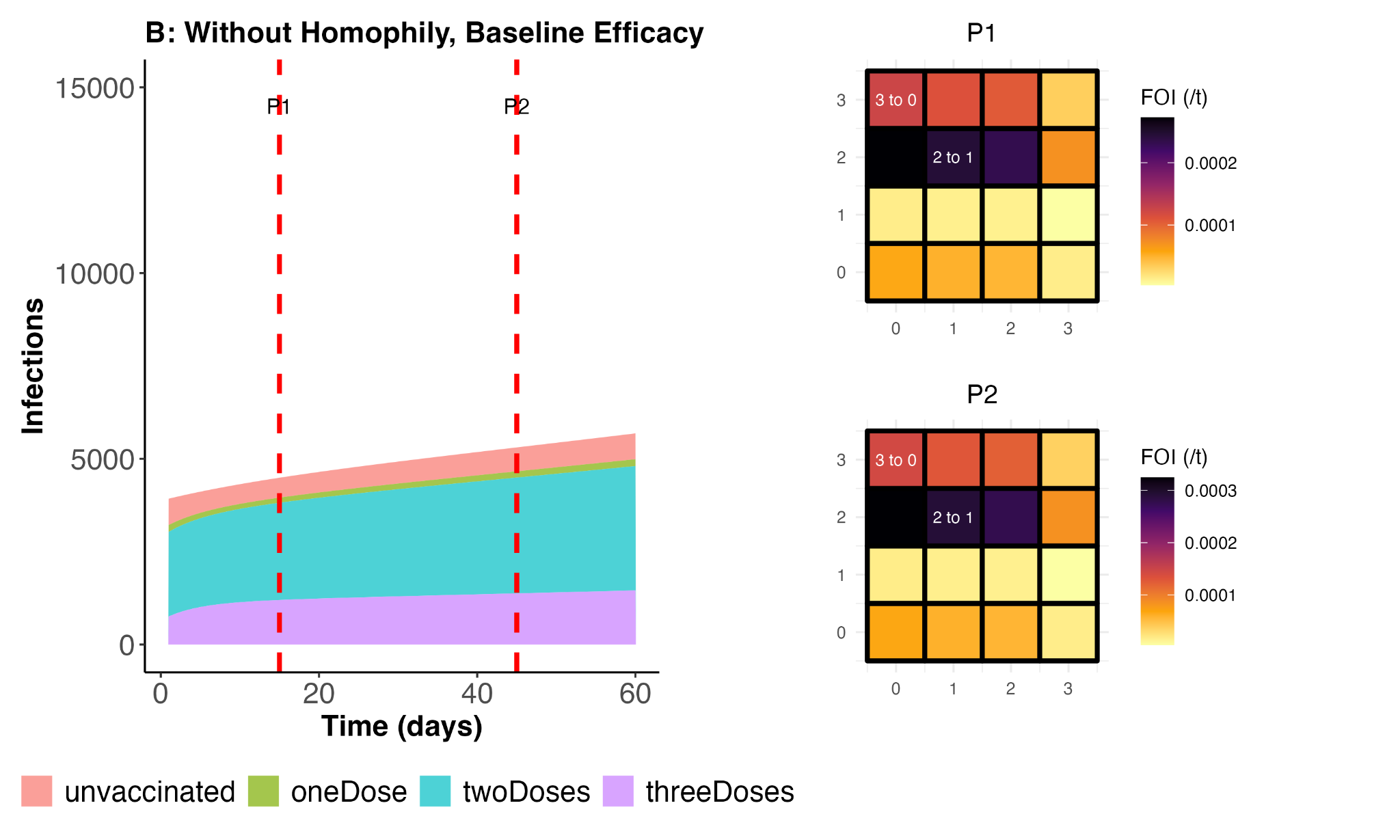


**Figure S4.** Scenario Showing Infections by Vaccination Status and Force of Infection Generated by Vaccination Groups on Days 15 and 45. The baseline parameter values are as follows (other parameters take the same values as in Figure 4 in the main text unless otherwise noted): $\tau_{0}$=0.35, $\tau_{1}$= 0.65, $\tau_{2}$=0.68, $\tau_{3}$=0.83 and $v_{0}=0, v_{1}=0.1,v_{2}=0.148,v_{3}=0.74$.

**
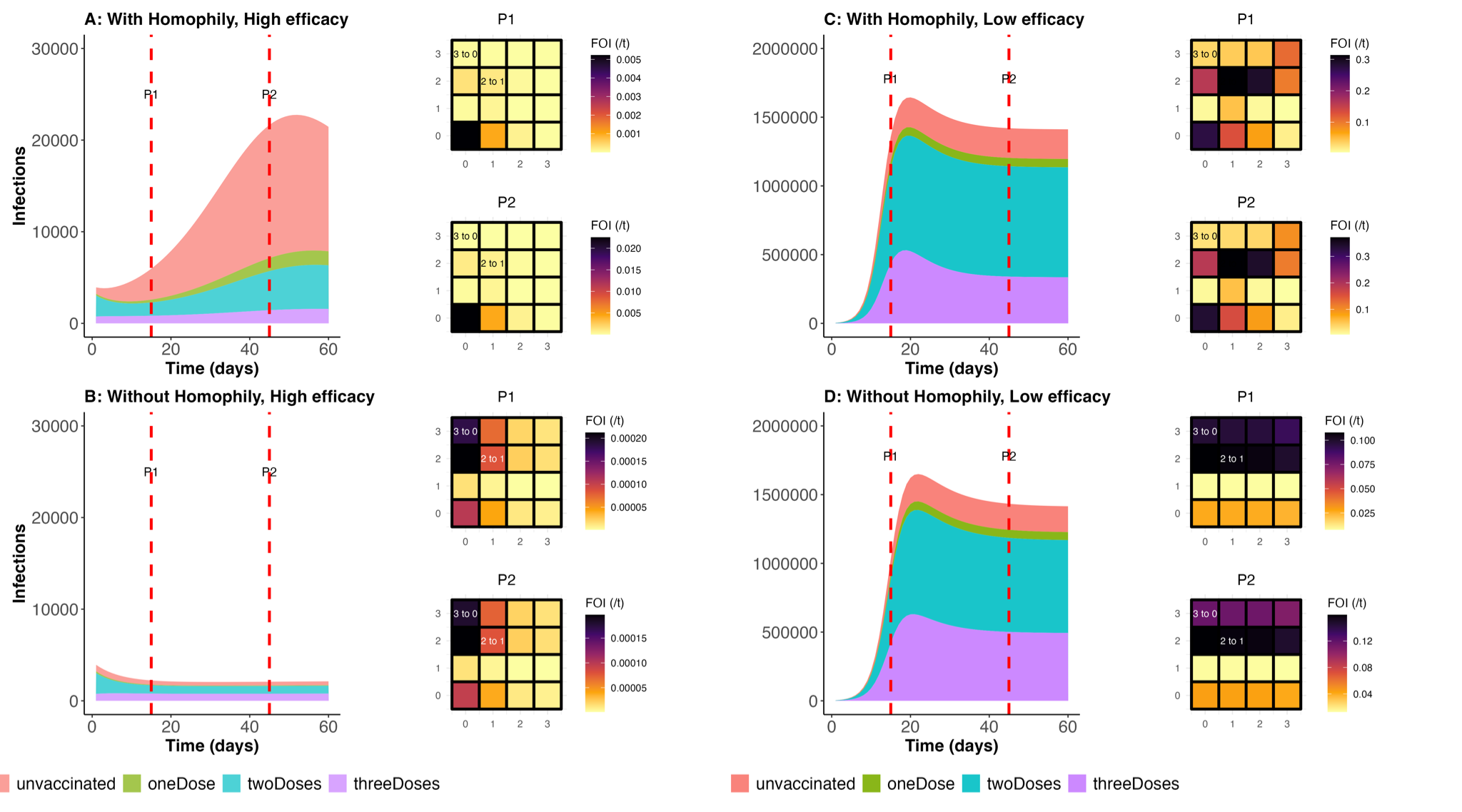
**

**Figure S5.** Scenario Showing Infections by Vaccination Status and Force of Infection Generated by Vaccination Groups on Days 15 and 45, where the level of adherence to public health measures is not part of the force of infection. Parameters take the same values as in Figure 4 in the main text.

**Table S1. Variables**

| **Variable** | **Measurement** |
| --- | --- |
| COVID-19 infection history | “Have you ever been diagnosed with COVID-19?” Participants could choose from the following responses:  (a) Yes, I have been diagnosed with COVID-19  (b) No, but I think I have had COVID-19. I just never received a test and/or diagnosis  (c) No, and I do not think I have had COVID-19 |
| Compliance with provincial mandates and guidelines | “On a scale of 1 (Not At All) to 4 (Very Closely), how closely have you followed provincial mandates and guidelines for COVID-19 prevention?” Participants could choose from the following responses:  (a) Not at all  (b) Not very closely  (c) Somewhat closely  (d) Very closely |
| Vaccination status | “How many doses of the COVID-19 vaccine have you received so far?” Participants could choose from the following responses:  (a) 0 doses  (b) 1 dose  (c) 2 doses  (d) 3 or more doses |
| Perceived COVID-19 history of regular contacts | “Thinking about the people you have regular contact with, how many of them have had COVID-19?” They were given the further instruction, “If you are unsure about how many have had COVID-19, provide your best estimate.” Participants could choose from the following responses:  (a) A few (i.e., 0%–20%)  (b) Some of them (i.e., 21%–40%)  (c) Around half of them (i.e., 41%–60%)  (d) Most of them (i.e., 61%–80%)  (e) Nearly all of them(i.e., 80%–100%) |
| Perceived compliance of regular contacts with COVID-19 prevention guidelines and mandates | “Thinking about the people you have regular contact with, how many of them have followed provincial guidelines for COVID-19 prevention ‘very closely’?”  (a) A few of them (i.e., 0%–20%)  (b) Some of them (i.e., 21%–40%)  (c) Around half of them (i.e., 41%–60%)  (d) Most of them (i.e., 61%–80%)  (e) Nearly all of them (i.e., 80%–100%)  They were given the further instruction, “If you are unsure about whether others are following guidelines ‘very closely,’ provide your best estimate.”  “If you had to provide an exact percentage, what percentage of those you have regular contact with are vaccinated?” |
| Vaccination status of household contacts | “Excluding yourself, how many people 12 years of age or older live in your household?”  The following clarification was also provided: “By household we mean anyone living at the same address as you, that you share a kitchen with.” |
| Number of people in their household that had received each level of vaccine dose | “For each person in your household, aged 12 years or older and excluding yourself, how many belong to each category.” The following clarification was also provided: “The total for the four categories below should sum to <<*household size provided above*>>, which you indicated was the number of people living in your household, excluding yourself.” Participants could choose from the following responses:  (a) 0 doses.  (b) 1 dose.  (c) 2 doses.  (d) 3 or more doses.  (e) Unknown |
| Vaccination status of non-household contacts | “The following questions are about the vaccination status of people you have been in contact with over the past 7 days, excluding members of your household. Please keep in mind the following as you answer these questions: COVID-19 is an airborne respiratory disease spread through saliva, other bodily fluids, and aerosols (such as those exhaled when talking, singing, sneezing, coughing, or breathing). Face-to-face contact, direct physical contact, and sharing the air of a person with COVID-19 in a poorly-ventilated indoor space increase your exposure to coronavirus-containing aerosols.” |
| Number of contacts | “In the past 7 days, how many people, regardless of whether they had COVID-19 or not, have you had contact with?” The following clarification was also provided: “Please do not include members of your household in this count. In providing this number, please use your best estimate. Please, include only people whom you interacted with directly.” |
| Number of contacts for whom they knew the vaccination status | “In the previous question, you reported that you had contact with <<*non-household contact network size provided above*>> people in the past 7 days. Of these people, how many do you know the vaccination status of?” Participants were reminded that, “This number should be the same or smaller than the number (i.e., <<*non-household contact network size provided above*>>) that you provided in the previous question. In the next question we will ask you to report on the vaccination status for these people.” |
| Number of people among their non-household contacts that had received each level of vaccine dose | , “For people who you were exposed to in the past 7 days, excluding members of your household, how many belong to each category? The following clarification was also provided: “The total for the four categories below should sum to <<*contacts whom participants knew the status of*>>, which you indicated was the number of people who you had been in contact with and whose vaccination status you knew.”  Participants could choose from the following responses:  (a) 0 doses.  (b) 1 dose.  (c) 2 doses.  (d) 3 or more doses.  (e) Unknown |
